# Hierarchical transcriptional network governing heterogeneous T cell exhaustion and its implications for immune checkpoint blockade

**DOI:** 10.3389/fimmu.2023.1198551

**Published:** 2023-06-16

**Authors:** Weihong Tian, Gaofeng Qin, Miaomiao Jia, Wuhao Li, Weili Cai, Hui Wang, Yangjing Zhao, Xuanwen Bao, Wangzhi Wei, Yu Zhang, Qixiang Shao

**Affiliations:** ^1^Department of Immunology, School of Medicine, Jiangsu University, Zhenjiang, Jiangsu, China; ^2^Life Science Institute, Jinzhou Medical University, Jinzhou, Liaoning, China; ^3^Jiaxing Key Laboratory of Pathogenic Microbiology, Jiaxing Center for Disease Control and Prevention, Jiaxing, Zhejiang, China; ^4^Institute of Medical Genetics and Reproductive Immunity, School of Medical Science and Laboratory Medicine, Jiangsu College of Nursing, Huai’an, Jiangsu, China; ^5^Department of Medical Oncology, The First Affiliated Hospital, School of Medicine, Zhejiang University & Key Laboratory of Cancer Prevention and Intervention, Ministry of Education, Hangzhou, Zhejiang, China

**Keywords:** T cell exhaustion, heterogeneous, T cell factor 1, chronic TCR stimulation, TOX, PD-1, immune checkpoint blockade

## Abstract

The fundamental principle of immune checkpoint blockade (ICB) is to protect tumor-infiltrating T cells from being exhausted. Despite the remarkable success achieved by ICB treatment, only a small group of patients benefit from it. Characterized by a hypofunctional state with the expression of multiple inhibitory receptors, exhausted T (Tex) cells are a major obstacle in improving ICB. T cell exhaustion is a progressive process which adapts to persistent antigen stimulation in chronic infections and cancers. In this review, we elucidate the heterogeneity of Tex cells and offer new insights into the hierarchical transcriptional regulation of T cell exhaustion. Factors and signaling pathways that induce and promote exhaustion are also summarized. Moreover, we review the epigenetic and metabolic alterations of Tex cells and discuss how PD-1 signaling affects the balance between T cell activation and exhaustion, aiming to provide more therapeutic targets for applications of combinational immunotherapies.

## 1 Introduction

Despite the tremendous clinical success of ICB therapy, the challenge brought by T cell exhaustion, which sustains immune tolerance, and limits the prolonged benefits of cancer immunotherapy, has raised great concerns about its linkage with poor clinical outcomes. Defined with decreased cytotoxic efficacy, diminished inflammatory cytokines secretion (e.g. IL-2, IFN-γ, and TNF), and elevated expression of suppressive immune checkpoints (such as PD-1, CTLA-4, LAG-3, TIM-3, TIGIT, and CD39), exhausted T (Tex) cells are dysfunctional clusters in hierarchical heterogeneity (1). Ranging from the stem-like stage (precursor Tex cells, Tpex) to the entirely function-lost stage (terminal Tex cells, Tex^term^), Tex cells are gradually developed with significant alterations in transcriptome and epigenome (2, 3).

The Tpex cells are capable of self-renewal and maintaining a chronically stimulated Tex cell pool (4). They are the main responder in mediating the therapeutic response of PD-1 blockade while the Tex^term^ cells are totally function-restrained (4–7). T cell exhaustion is an inevitable outcome initiated by sustained TCR signaling under long-lasting inflammation (8). Nevertheless, this program protects Tpex against excessive differentiation or over-stimulation-induced apoptosis (9). This mechanism partly explains why serious autoimmunity is a side effect of ICB treatments even though interfering with exhaustion contributes to therapeutic benefits.

Tex cells exhibit altered transcriptomes compared to effector T (Teff) cells and memory T (Tmem) cells. Transcription factor (TF) NFAT and NR4A, downstream of persistent TCR signaling, play vital roles in initiating T cell exhaustion (10). A transcriptional hierarchy of coordinated TF networks regulates the differentiation of distinct Tex clusters. The feedback loop of T cell factor-1 (TCF-1)-thymocyte selection-associated high mobility group box protein (TOX) and T-box family members is critical to control the development of T cell exhaustion (10–12). Instead of causing a temporary reduction of T cell function, exhaustion is a unique and epigenetically fixed state which is irreversible during T cell development. Tex cells show decreased chromatin accessibility of effector-related genes but increased accessibility of genes encoding inhibitory receptors (IRs). Observations indicate that Tex cells could only be transiently rejuvenated by anti-PD-1 and they will ultimately tend to terminal exhaustion (13). Targeting epigenetic modifications of Tex cells are supposed to be taken into consideration sufficiently to improve immunotherapies.

Efforts to overcome the limitations of ICB have focused on better understanding the molecular mechanisms underlying T cell exhaustion, as well as the transcriptional interactions between Tex cells and immune checkpoint receptors and ligands. In this review, we elucidate developmental pathways and transcriptional regulation of Tex cells. We aim to examine the cellular and molecular machinery that governs Tex formation, differentiation, and response to PD-1 blockade. Additionally, we discuss therapeutic strategies aimed at restoring the effector functions of Tex, which may ultimately improve the clinical response to immune checkpoint blockade.

## 2 The heterogeneity of exhausted T cells in a differentiation hierarchy

**Figure 1:**
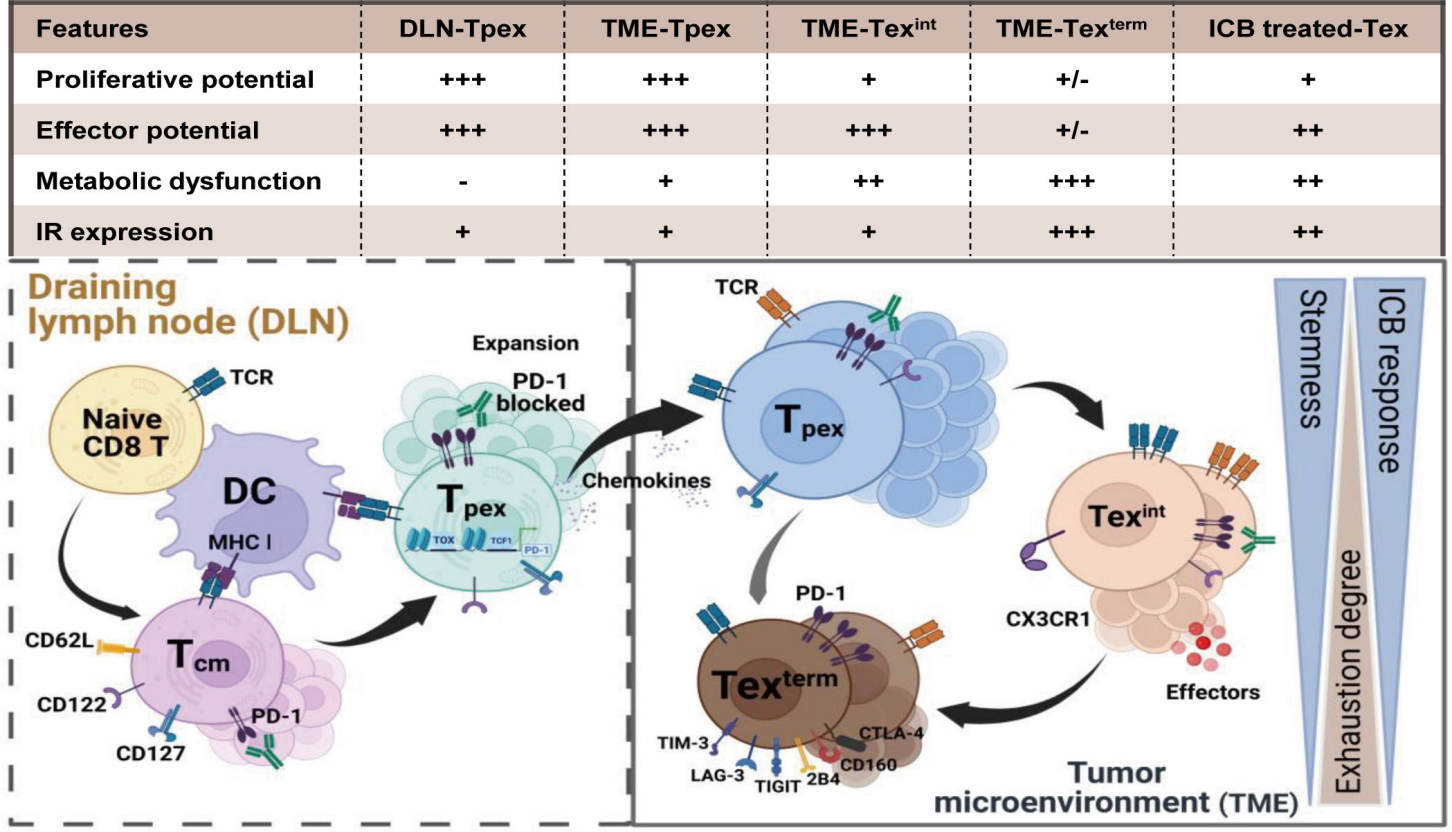
The differentiation hierarchy of exhausted T cells. The exhaustion program of Tex cells follows a hierarchical differentiation process with progressive loss of effector function and proliferative capability. Activated by tumor antigen, naïve CD8^+^ T cells differentiate into central memory T cells (Tcm). However, persistent stimulation of tumor antigens triggers Tcm cells to form the function-restrained progenitor Tex cells (Tpex). Tpex cell cluster preferentially localizes in lymphoid organs and is responsive to PD-1 blockade. Once migrating into the TME, the Tpex cluster subsequently differentiates spanning from the effector-like intermediate Tex cells (Tex^int^) to the terminal Tex cells (Tex^term^) for maintaining the anti-tumor T cell pool. Persistent TCR signaling and suppressive co-stimulatory signals in the TME force the development of T cell exhaustion. Tpex cells gradually lose the proliferative potential while converting to progeny Tex cells. The expression of inhibitory receptors (e.g. PD-1, LAG3, TIM3 and TIGIT) elevates in terminal exhausted T cells. Accompanied by metabolic dysfunctions and epigenetic modifications, the exhaustion state of Tex^term^ cells is irreversible and they can minorly respond after PD-1 blockade. Created with BioRender.com.

Exhausted T cells exhibit a hierarchical cell differentiation trajectory (**Figure 1**). Naïve CD8^+^ T cells differentiate into cytotoxic effector cells and memory cell precursors after cell activation (14, 15). While in chronic infections and tumors, an early Tpex cell cluster develops and preferentially localizes in lymphoid organs (4, 7). This Tpex cluster is proliferative and function-reserved. Tpex cells subsequently generate additional subsets of Tex cells containing the transitory Tex clusters and the terminal Tex cell clusters (2, 16, 17) in chronic infections and tumor microenvironments (TME), reflecting a cell differentiation hierarchy necessary for maintaining the functional T cell pool. This progressive development of T cell exhaustion results in a high heterogeneity of Tex cells. We discuss the heterogeneity of Tex cells and the epigenetic modifications underlining this stepwise differentiation in the following.

### 2.1 Phenotypic and functional heterogeneity of Tex cells

The heterogeneity of Tex cells mainly depends on their distinguished phenotypes, effector molecules, key transcription factors and inhibitory receptors. Four heterogenous Tex subpopulations with developmental relationships were identified during chronic viral infections and tumors (18). There existed two progenitor Tex (Tex^prog^) subpopulations, Tex^prog1^ and Tex^prog2^, which shared related open-chromatin landscapes but differed in phenotypes and functions. Tex^prog1^ upregulated genes associated with stem-cell biologies, such as *Tcf7*, *Myb*, *Il7r* and *Sell*. While genes preferentially up-regulated in Tex^prog2^ were related to cell cycle (*Cdk* and *Mki67*) and cell migration (*Anxa2*, *Itgb7* and *Alcam*). Tex^prog1^ was resident and capable of transforming into Tex^prog2^, which could re-localize from blood-inaccessible lymphoid niches to blood-accessible locales. The Tex^prog2^ subpopulation underwent a proliferation-driven transition to the intermediate Tex (Tex^int^) cells, which presented as a transitional population with some cytolytic and migratory capacities. The transcriptional characteristic of the Tex^int^ population contained genes linked to cytotoxic functions, such as *Gzma*, *Gzmb*, *Prf1*, *Klrk1* and *Cx3cr1*. Tex^int^ also expressed higher TF genes like *Tbx1*, *Zeb2* and *Id2*. The Tex^term^ subpopulation exhibited the most severe exhausted profile and the weakest cytotoxicity. Tex^term^ up-regulated multiple inhibitory receptor genes (e.g., *Pdcd1*, *Lag3*, *Tigit* and *Cd244*) and molecule genes related to terminal depletion (*Entpd1*, *Cd101* and *Cd38*). Meanwhile, the Tex^term^ showed elevated TCR signalings with highly expressed *Zap70* and *Nfatc1*. Moreover, although the key exhaustion regulator TOX (19, 20) was expressed by all Tex cells, the Tex^term^ population exhibited the highest expression.

The differentiation hierarchy of Tex cells confirms the progressive development of T cell exhaustion. Within the spectrum of T cell exhaustion, numerous Tex cells are identified. Zhang et al. identified five main pan-cancer Tex clusters across 30 bulk solid tumor types (21). These five Tex clusters in the TME showed Tex-specific profiles in glycolysis, chemokinesis and cytotoxic functions. The transcriptional factor TCF-1 and T-bet gradually decreased among the five clusters from Tex^prog^ to Tex^term^. TOX had a decreasing trend from Tex^prog^ to Tex^int^ but was highest in the Tex^term^. Besides, the Tex^term^ subset displayed reduced gene mutations and DNA methylations when comparing the genomic alterations. Similarly, Bengsch et al. also uncovered nine distinct Tex clusters in human HIV infection and lung cancer by using a comprehensive mass cytometry approach (22). Validated by a functional exhaustion score, the Tex clusters with higher scores had elevated co-expression of IRs and TF Eomesodermin (Eomes). Conversely, a lower score owned Tex clusters expressed higher levels of functional markers like CD127 and TCF-1 but lower levels of IRs like PD-1, CD38 and 2B4. Additional observations in patients with B-ALL also illustrated that Tex cells are remarkably heterogeneous in cell cycle and expression patterns of cytotoxic molecules (23).

Totally, the expression of IRs like PD-1, CTLA-4, LAG-3 and TIM-3 is considered a crucial factor for categorizing Tex cells. The PD-1^lo^ subset expressed fewer or no IRs such as TIM-3 and 2B4, and can differentiate into the PD-1^hi^ subset (24). TCF-1 plays a crucial role as a central transcription factor in the PD-1^lo^ Tex cluster (12, 25–27). The TCF-1^+^PD-1^lo^ Tex cluster exhibits a self-renewing capacity and maintains persistent T cell responses (18, 21, 22, 28, 29) in chronic infections and cancers. Based on the expression intensity of IRs and TCF-1, Tex cells are generally divided into three stages (1): the Tpex, which is the stem-like PD-1^lo^TCF-1^+^ Tex precursors (2); the Tex^term^, which is the terminally differentiated PD-1^hi^TCF-1^-^ Tex cluster with the highest exhaustion profile; and (3) the Tex^int^, which is at a transitional stage between the Tpex and Tex^term^, characterized by intermediate PD-1 expression but without TCF-1 and has the highest effector functions.

### 2.2 Epigenetic remodeling in the development of T cell exhaustion

Epigenetic studies have shown that exhaustion is a unique and enduring stage in T cell differentiation rather than merely a momentary reduction of T cell function. Approximately 6,000 open chromatin regions distinguish CD8^+^ Tex cells from effector and memory T cells (30, 31). The exhaustion program is firmly established in the epigenome of Tex cells. Although the inhibitory receptor has been blocked, the epigenetic remodelings of Tex cells are minorly changed (30). This explains why reinvigorating PD-1^lo^CD8^+^ Tex cells can merely cause a transient cytotoxic activity in response to ICB therapy.

Tex cells exist within a range of steady and constant epigenetic landscapes. In a general model, exhaustion-regulated genes are accessible in Tex cells and are transcriptionally controlled by exhaustion-related transcription factors. Memory-related genes are inaccessible to the transcriptional machinery, whereas several effector-related genes are still available in exhausted T cells but are not effectively bound by transcription factors. For example, the chromatin-accessible regions at the *Ifng* and *Gzmb* locus both emerged in CD8^+^ Teff cells and Tex cells, but the gene expressions were significantly distinct on the basis of functional pathway enrichment (31). Tex cells exhibited significant alterations in the regulation of IFN-γ production and immune effector process pathways (31). However, in reinvigorated Tex cells by PD-L1 blockade, the exhaustion relevant genes like *Pdcd1* and *Ctla4* are still expressed for DNA demethylation of these gene loci. Although inhibition of PD-1 signaling activated the expression of many effector genes like *Gzmb* and *Ifng*, the revived Tex cells eventually returned to be exhausted under consistent high antigen stimulation in chronic LCMV infection. The anti-PD-L1 treated Tex cells were unable to differentiate into Tmem cells even if the antigen was eliminated (30). This suggests that the powerful reinvigoration of checkpoint blockade may not be long-lasting without any epigenetic alterations.

Via transposase-accessible chromatin sequencing (ATAC-Seq), CD8^+^ Tex cells reveal numerous distinctive genomic features. A Nr4a1 binding motif at -22.4 kb of the murine *Pdcd1* locus becomes accessible in Tex cells (32), and a *de novo* enhancer at approximately 23.8 kb upstream the transcription start site of *Pdcd1* is also identified (30, 31). Functional analysis of this enhancer has shown that it is necessary for the persistent and integrated expression of PD-1 in Tex cells (31). Epigenetic modifications at the *Pdcd1* locus reflect the regulatory differences between Tex and Teff cells. During the early phase of chronic infection, the differentiation of CD8^+^ Teff cells is followed by a temporal loss of DNA methylation at the *Pdcd1* locus (33). Following durable TCR signaling, histone acetylation facilitates the opening of *Pdcd1* enhancer elements. Some of these gene loci are found to be recognized by T-bet, directly supporting that T-bet also functions to regulate PD-1 expression (34). In exhausted CD8^+^ T cells, the *Pdcd1* region is entirely demethylated and it remains in this status even when inflammation is eliminated. The absence of DNA methylation leaves the *Pdcd1* locus primed for accelerated expression (35).

It is crucial to identify gene loci that are epigenetically modified and related to severe T cell exhaustion for improving immunotherapies. Zhang et al. demonstrated that treating TILs with valproic acid, a histone deacetylase inhibitor, *ex vivo* reduced the exhaustion degree of Tex cells and improved the therapeutic effect of ICB treatment (36). Additionally, Ghoneim et al. found that Dnmt3a mediates the DNA methylation reprogramming in the development of terminal T cell exhaustion and targeting Dnmt3a synergizes with PD-1 blockade to improve ICB therapy (13). To develop new therapeutic options, an in-depth understanding of the molecular mechanisms underlying epigenetic modification involved in T cell exhaustion is needed.

## 3 Hierarchical transcriptional regulations of T cell exhaustion

**Figure 2:**
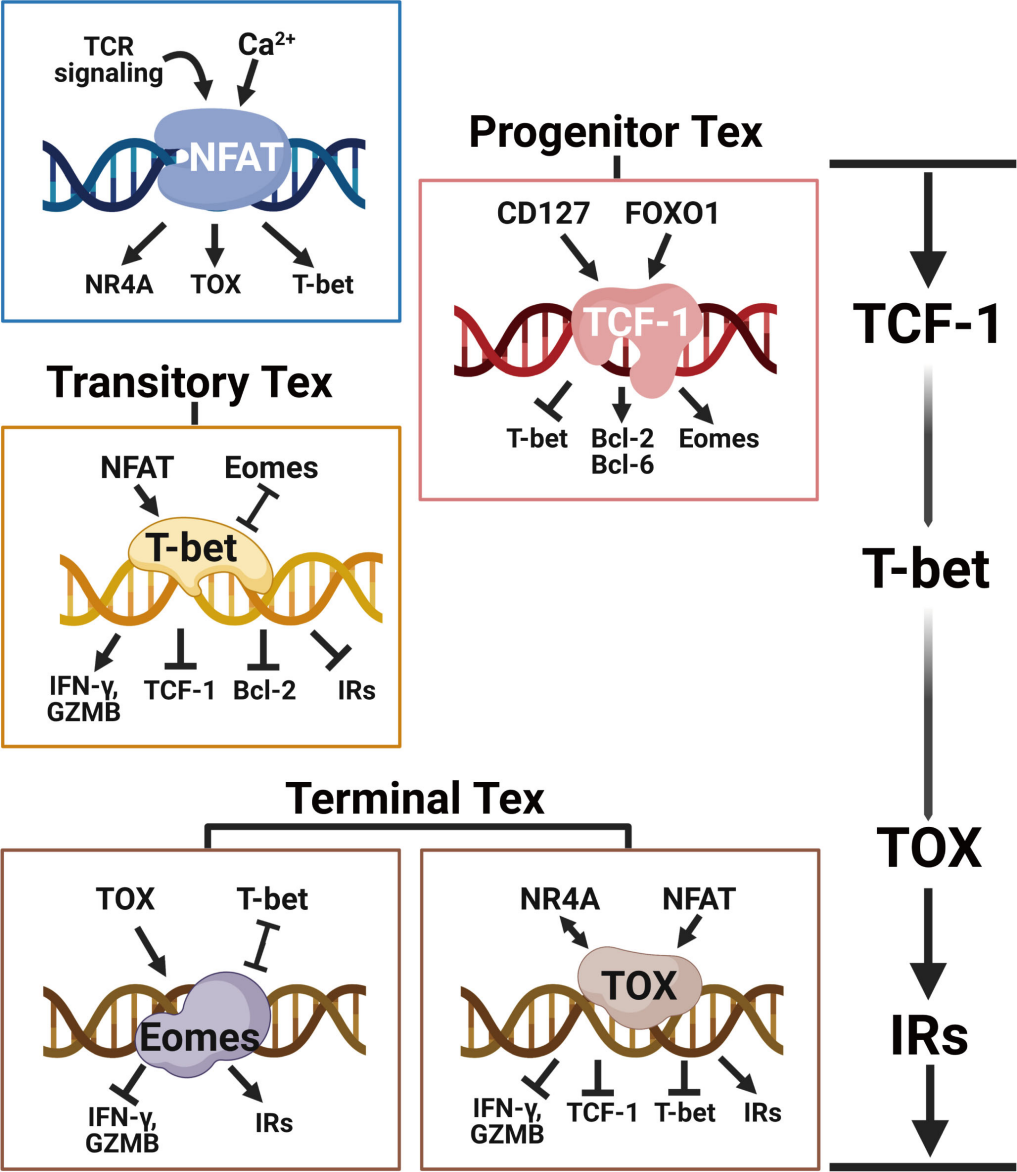
The transcriptional hierarchy of regulating T cell exhaustion. Following continued TCR stimulation and downstream Ca^2+^ signaling, the transcriptional network orchestrated by NFAT directly induces the expression of TOX and NR4A to initiate the T cell exhaustion program. TCF-1 is central for maintaining the stemness of Tpex cells by enhancing Eomes and Bcl-2 expressions. With the ongoing NFAT signaling, T-bet counterbalances TCF-1 and Eomes to facilitate the expression of effector molecules like IFN-γ and granzyme B in transitory Tex cells. The eventually upregulated TOX and Eomes prevent T-bet-mediated effector programming and induce the expression of multiple inhibitory receptors (e.g. PD-1, LAG-3, TIM-3, 2B4 and TIGIT), epigenetically imposing the terminal T cell exhaustion program. Created with BioRender.com.

Exhausted T cells exhibit altered transcriptional profiles. Key TFs in regulating heterogeneous Tex clusters display different expression patterns of concentrations and subcellular localizations, and they also show distinct downstream interactions with specific co-factors and accessible chromatins (37). Importantly, a transcriptional hierarchy of coordinated TF networks exists to establish and regulate the exhaustion program (38). Under continuous TCR signaling, Tex cells undergo a hierarchical differentiation trajectory from the Tpex cells to the transitory Tex cells, eventually to the Tex^term^ cells. The critical transcription factors TCF-1, TOX, and T-box family members T-bet and Eomes involved in varying regulation networks take turns to modulate the exhaustion program of different Tex clusters (**Figure 2**).

### 3.1 TCR signal strength in initiation of T cell exhaustion

T cell exhaustion is initially driven by persistent and high amounts of TCR stimulation in chronic infections or tumors (39). The calcium influx from TCR signaling promotes the activation of TFs NFAT, BATF and IRF4 (40). This TCR-responsive TF network is crucial for maintaining T cell expansion and function (41–43). Associated with the antigen loads, T cells express higher levels of these TFs in chronic infections than in acutely solved infections (44). At the early stage of T cell activation, NFAT translocates into the nucleus to form heterodimers with AP-1 for fostering the expression of T cell effector genes (45, 46). However, at the late effector stage, the ratio of nuclear NFAT : AP-1 was abnormally risen (46, 47). On the one hand, the consistent TCR signaling recruits superfluous NFAT to relocate into the nucleus, resulting in a higher ratio of NFAT : AP-1 and leaving many NFATs free. On the other hand, the increased BATF signaling antagonizes the formation of classic AP-1 complex, leading to less AP-1 and a much higher NFAT : AP-1 ratio (42, 48). NFAT is reported to function as an effective PD-1 promoter (46) and elevate the expression of NR4A and TOX (49). As a result, the excessively free NFATs primarily promote the expression of inhibitory receptor genes of T cells, and gradually drive the acquisition of exhausted phenotypes (11, 49, 50)

What’s more, the TCR signal strength determines the differentiation of Tex cells. It has been observed that the T cell cluster specific for antigen with lower affinity, compared with another cluster recognizing antigen with higher affinity, is more abundant in Tpex cells in the same tumor (51). Besides, Daniel et al. found that high TCR signaling correlates with more terminal Tex cells, whereas low TCR signal strength generates the effector-like Tex cell cluster with a killer cell lectin-like receptor (KLR)-expressed cytotoxic profile (52). Shakiba et al. also demonstrated that tumor-specific CD8^+^ T cells, whether undergoing low or high TCR signal strength, both upregulated inhibitory receptors and couldn’t mediate tumor control. However, high TCR signaling leads to an effector function loss and a dysfunctional state of CD8^+^ T cells while the low TCR signal restrains them in a functional state (53). These findings all illustrate that TCR signal strength is a key determinant of T cell response and Tex cell formation.

### 3.2 The TCF-1-TOX-T-Box signaling feedback in hierarchical regulations of Tex cells

#### 3.2.1 Transcriptional circuit of TCF-1-TOX and Eomes in maintaining progenitor Tex cells

Progenitor Tex cell cluster generates early during chronic infections and they are able to proliferate and self-renew for their expression of TCF-1 (38). TCF-1 signaling plays a crucial role in generating Tpex cells during persistent inflammation by antagonizing the Teff cell-driving TFs like T-bet and ID-2 (4, 12). In early bifurcation of T cell differentiation, TCF-1 represses the developmental path to the terminal KLRG1^hi^ Teff cells and promotes the formation of the Tpex cluster (12, 40). Studies have shown that ectopic TCF-1 expression strongly promotes the formation of Tpex in both chronic infection and pre-clinical tumor models (26). Adoptive transfer experiments have similarly demonstrated that TCF-1^+^ Tex cells are capable of proliferating and maintaining T cell responses. In contrast, Tex cells that have lost TCF-1 expression are unable to proliferate and maintain T cell responses (4, 7). Furthermore, TCF-1 overexpression diminishes the expression of co-inhibitory receptors and enhances cytokine production capacity (54). TCF-1^+^ Tpex population is the main cluster of the proliferative burst after PD-1 blockade, indicating their retained functions in comparison to other Tex clusters (4).

TOX is a pivotal regulator of T cell exhaustion (19, 20). In chronic infections and tumors, TOX can be induced by TCR signaling quickly and keeps high expression specifically in the Tex cell lineage. But in acute infections, TOX is transiently induced and expressed at low levels with no persistence (11, 49, 55). NFAT signaling downstream of TCR stimulation activates *Tox* gene expression directly (55). The expression of TOX increases the production of PD-1 and other inhibitory receptors (55, 56), establishing the exhaustion phenotype of early exhausted T cells. The deletion of *Tox* abrogated T cell exhaustion program in chronic viral infection. In *Tox*-deficient tumor-infiltrating T cells, a large fraction of gene loci for inhibitory receptors like *Pdcd1*, *Havcr2* and *Tigit* are revealed to be inaccessible (11). However, the *Tox*-deficient T cells preserved high expression of TCF-1 (11). Indeed, at the early stage of LCMV infection, the TOX^+^ T cell subset positively connected with TCF-1 expression (55). It was reported that KLRG1^+^ terminal Teff was unable to generate TOX^+^ T cells, perhaps due to their lack of TCF-1 (57, 58).

The T-box transcription factors Eomes and T-bet are both indispensable for the development of T cell exhaustion. During early chronic infections, TCF-1 antagonizes T-bet and promotes a T-bet to Eomes conversion in precursor Tex cells by increasing Eomes expression, which controls a Bcl-2 mediated Tex cell survival (12). Eomes promotes IL-15Rβ expression and T cell memory formation during acute infections (59, 60), while T-bet is crucial for effector function and drives KLRG-1^+^ Teff cells formation. Eomes is straightforwardly connected with the TCF-1 expression in naive and memory CD8^+^ T cells. Actually, enhanced expression of Eomes partly saves TCF-1-deficient memory CD8^+^ T cells from progressively losing (27). The acquisition of memory features in Tpex is then promoted by the activation of *Eomes* gene transcription, which is necessary to maintain the Tex cell pool (26).

In conclusion, at the early stage of chronic infections and tumors, probably within the first seven days of chronic antigen stimulation (61), TCF-1, under control of TCR and PD-1 signaling, fosters Eomes expression in Tpex to blunt the activity of T-bet, antagonizing terminal Teff cell differentiation. TCF-1 signaling allows adequate time for TOX-dependent epigenetic remodeling in stepwise fixing the exhaustion program of Tpex cells (20). Importantly, the TCF-1^+^ Tpex cells are responsible for the proliferative boost after immune checkpoint blockade (4, 62), and the proportion of this cluster in tumors correlates positively with advanced clinical outcomes (7, 63, 64). In this way, the Tpex cell cluster is potential to perform as a therapeutic marker for ICB therapy clinically.

#### 3.2.2 Transcriptional role shifts from Eomes to T-bet in regulation transitory Tex cells

The transitory Tex cell cluster shows enrichments of T-bet and RUNX motifs in ATAC-seq analysis of TF motif accessibility, while the Tex^term^ cluster shows enrichments of NR4A and Eomes motifs (18). For one thing, it implies a transcriptional signaling shift of T-box family members whose activity may emphasize distinct Tex clusters when differentiation. For another thing, it indicates that T-bet and Eomes have separate functions in chronic infections and cancers from their roles in Teff and Tmem cells during acute infections (65).

The transitory Tex cell cluster is effector-like and exists at an intermediate cell state between Tpex cells and Tex^term^ cells. T-bet counterbalances TOX to stabilize the transitional exhaustion state and prevent terminal exhaustion. T-bet inhibits the transcription of Pdcd1 and possibly other immune checkpoint genes (34) while Eomes expression positively correlates with the expression of PD-1 and other IRs in Tex cells (22, 66). Differential nuclear localization of T-bet and Eomes regulates the T cell exhaustion program. T-bet and Eomes compete for binding to the same DNA motifs that contain the Pdcd1 locus. A decreased nuclear Eomes in Tex cells allows T-bet to play a dominant role as a stronger inhibitor of Pdcd1. Silencing PD-1 signaling in Tex cells increases T-bet nuclear localization, which initiates the expression of T-bet-related functional genes for chemotaxis, homing, and activation (67).

Dynamically, Eomes is higher in the Tpex cluster for TCF-1 activation whereas it declines in the transient Tex^int^ cluster. T-bet reaches its highest level in the Tex^int^ cluster before dropping at the Tex^term^ stage (18). Most notably, although researches have shown that transient Tex cells give rise to Tex^term^ cells, there still has evidence that transitory Tex cells may serve as the endpoint of Tex cell differentiation based on the TCR lineage tracing analysis (16, 68). One possible explanation of this finding could be the heterogeneity of transitory Tex cells. The Tex^int^ cells are also heterogeneous and could progress through a CX3CR1^+^ KLR^+^Tex cell state which represents an alternative terminus of Tex cell differentiation (52).

#### 3.2.3 Functions of TOX and Eomes in commitment to terminal T cell exhaustion

Terminal exhausted T cells derived from transitory Tex cells are defined by their high expression of TOX and multiple IRs like PD-1, LAG-3, TIM-3, CD38 and CD101 (2, 18). Persistent TCR and NFAT signalings induce *Tox* expression adequately. The endurable TOX signal interacts directly with histone acetyltransferase and indirectly with DNA methyltransferases to epigenetically fix CD8^+^ Tpex cells towards terminal exhaustion (20, 69). Eomes expression is higher in the Tex^term^ cluster (67). Eomes synergizes with TOX at the late stage of chronic infection to promote more severe T cell exhaustion (18). The higher expression of Eomes in the Tex^term^ subset is distinct from its expression in the quiescent self-renewing Tmem cells during acutely resolved infections. Eomes partially antagonizes T-bet and determines the Tex profile through greater nuclear localization and higher affinity to Pdcd1 binding sites. Eomes is higher in the Tpex cluster for TCF-1 activation and it declines in the transient Tex^int^ cluster while rebounding at the Tex^term^ stage.

With consecutive antigen exposure, Tex cells eventually proceed to the Tex^term^ cluster with elevated expression of TOX and Eomes. These two key transcriptional factors orchestrate other exhaustion regulators like NR4A to promote the terminal exhaustion program that includes highly expressed inhibitory receptors, decreased effector functions and elevated metabolic alterations.

## 4 Metabolic regulations of T cell exhaustion

**Figure 3:**
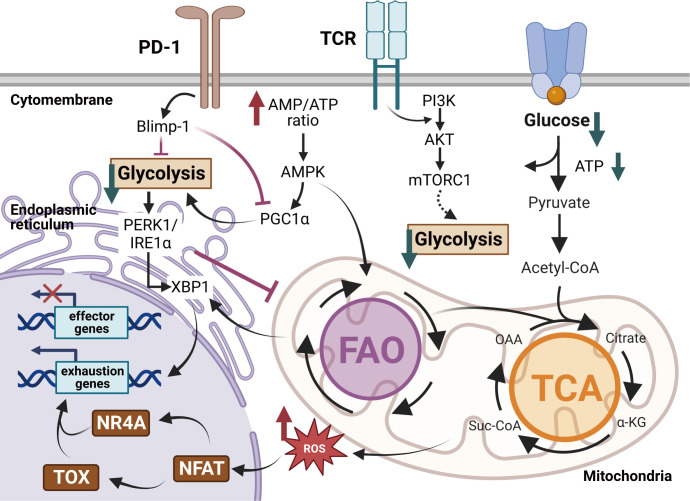
Metabolic regulations of exhausted T cells in the TME. Exhausted T cells exhibit metabolic insufficiency with inhibited glycolysis, mitochondrial respiration and endoplasmic reticulum (ER) function. Enhanced PD-1 signaling in Tex cells dampens the transcriptional coactivator PGC1α, decreasing glycolysis but promoting fatty acid oxidation (FAO). The overloaded cholesterol elicits more ER stress via the XBP1 cascades and fosters the expression of inhibitory receptors. Moreover, impaired glycolysis activates the IRE1-XBP1 signaling pathway, resulting in elevated ER stress and dysregulations of mitochondrial function. The dysfunctional mitochondria produce robust ROS, ultimately promoting TOX expression and repressing effector functions in Tex cells. Created with BioRender.com.

Exhausted T cells display metabolic dysregulations in diminished glycolysis, impaired mitochondrial fitness and enhanced endoplasmic reticulum (ER)-related stress (**Figure 3**). The dysregulated metabolism profile also contributes to T cell exhaustion in long-lasting inflammations. Hypoxia, low pH and insufficient nutrients are representative conditions of TME to play active roles in T cell exhaustion. It was found that the cytolytic activity of CD8^+^ T cells was regulated partly by the hypoxia-inducible factors (HIFs) and the von Hippel-Lindau (VHL) tumor suppressor protein during chronic infections (70, 71). The VHL-deficient cytotoxic T lymphocytes exhibited improved control of persistent viral infection and tumor growth for their enhanced response to hypoxia (70). Impaired aerobic glycolysis strongly dampens T cell activation and cytokine secretions (72). Limited glucose and downregulated transporter GLUT1 result in poor glucose usage and accelerate the exhaustion program of Tex cells (73, 74).

Faulty metabolic reprogramming and altered mitochondrial responses lock T cells in the exhausted state (73, 75). Signalings of inhibitory receptors trigger metabolic insufficiency, mitochondrial dysfunctions and endoplasmic reticulum (ER)-related stress in Tex cells (76). The PD-1^+^ Tex cells exhibit abnormal metabolic programs in reduced glycolysis, protein glycosylation and oxidative phosphorylation (OXPHOS) (77, 78). The PD-1-related mitochondrial dysfunction follows the downregulation of the transcriptional coactivator PGC1α, which is essential for maintaining mitochondrial activity (79). Overexpression of PGC1α promotes mitochondrial fitness and maintains effector functions of tumor-infiltrating CD8^+^ T cells. Dysfunctional mitochondria result in a strong production of mitochondrial reactive oxygen species (mtROS), which facilitates T cell exhaustion by enhancing inhibitory receptors expression and impairing effector function genes (80). ER-related stress upregulates IRE1-XBP1 signaling, leading to the over-expression of inhibitory receptor genes like *Pdcd1* and *Cd244* (81). XBP1 signaling cascades repress the expression of *Tbx21* (82), which impairs T cell function. The mitochondria-ER contacts play an important role in T cell exhaustion. The metabolic interactions between mitochondria and ER are controlled by Ca^2+^ flux, mitochondria ROS (mtROS) and mitochondria-associated membranes (MAMs). MAMs have been demonstrated to act as critical immunometablic molecules that participate in metabolic reprogramming and secretion of IFN-γ in CD8^+^ T cells. Abnormal Ca^2+^-NFAT signaling coupled with the accumulation of mtROS under persistent TCR stimulation disrupts mitochondrial metabolism homeostasis and damages T cell survival.

The epigenetic exhaustion alterations are the major regulatory mechanisms linked with mitochondrial disorders and nuclear reprogramming (83). Metabolic insufficiency and dysfunctional mitochondrial activity increase epigenetic modifications in T cell exhaustion. Limited availability of glucose catabolism acetyl-CoA alters the chromatin accessibility to histone acetylation at *Ifng* and *Tbx21* gene loci. Under glucose restriction, acetate treatment speeds up histone acetylation and reduces *Ifng* gene accessibility, restoring IFN-γ secretion in CD8^+^ T cells (84). Moreover, NAD-dependent histone deacetylation accelerates T cell exhaustion in reconstructing chromatin structure by suppressing deacetylase sirtuins (SIRTs) activity. The activity of SIRT1 involves affecting IFN-γ production and the expression of *Tbx21* genes in CD8^+^ Teff cells (85). It is also reported that S-adenosylmethionine (SAM) contributes to the exhausted epigenetic remodeling during Tex cell differentiation (73). SAM metabolism is inferred to modulate DNA methylation programs on *Tcf7*, *Tbx21*, and *Ifng* gene loci (13) that are engaged in the differentiation of progenitor Tex cells.

Fine-tuning the metabolic activity of exhausted T cells improves T cell effects and responds to ICB treatment. PD-1 blocking during chronic LCMV infection reinstates mitochondrial fitness and boosts glucose absorption in Tex cells (77). The blockades of glutamine and acetate relieve certain metabolic dysfunctions and prevent T cell exhaustion in the TME (86). Promoting fatty acid oxidation with chemokine treatments and peroxisome-proliferator-activated receptor agonists improves CD8^+^ T cell tumoricidal effects (87). In addition, the IRE1 and PERK inhibitors effectively reduce ER stress and regain T cell functions. Closely monitoring the mitochondrial activity and controlling the metabolic demands seem to be efficient ways to overcome T cell exhaustion.

## 5 Co-stimulatory molecules, cytokines and kinases in T cell exhaustion

Factors and signaling pathways involved in the development of T cell exhaustion are diversified and complex. Besides the TCR signal strength, key transcriptional pathways and metabolic alterations mentioned above, the co-stimulatory signals and soluble mediators also widely participate in the progress of T cell exhaustion. We summarize the intrinsic and extrinsic factors contributing to T cell exhaustion in four general signal models in **Table 1**. And in the following, we discuss the co-stimulatory molecules, cytokines and kinases in T cell exhaustion.

**Table 1:** Summary of signal models in development of T cell exhaustion.

Signal model	Factor	Affect	Combinational therapies
Signal 1, (core) Persistent TCR signalling	Overloaded and persistent virus and/or tumour antigen	Excessive T cell activation	PD-1/PD-L1 blockade + OX40/4-1BB agonist
	Impaired co-stimulation	Decrease T cell function (CD27, CD28, OX40, 4-1BB, …)	
Signal 2, Co-inhibitory signals	Immune checkpoints	Inhibit T cell function (PD-1, 2B4, LAG-3, TIM-3, CD160, CTLA-4, …)	PD-1/PD-L1 blockade + CTLA-4/LAG-3/TIM-3/TIGIT/blockade
	Regulatory or suppressive immune cells	Inhibit T cell function (Tregs, MDSCs, …)	
Signal 3, Soluble mediators	Inflammatory cytokines	Induce the secretion of negative cytokines (IL-2, IFN-α/β, …)	PD-1 blockade + IL-2/IL-21 administration or IL-10R/TGF-βR blockade
	Suppressive cytokines	Attenuate T cell function (IL-10, TGF-β, TNF, …)	
Signal 4, Tissue or tumour microenvironment	Hypoxia	Metabolism reprogramming	PD-1 blockade + Chemotherapy or PPARs/PERK antagonist
	Low pH		
	Insufficient nutrients		
	Dysfunctional stromal cells	Recruit MDSCs to impair T cell function/Express multiple ligands of inhibitory receptors	PD-1/PD-L1 blockade + Chemotherapy or EGFR/VEGFR/HER2 antagonist
	Abnormal adhesion molecules	Suppress T cell function (cadherins, selectins, integrins, …)	

MDSCs, myeloid-derived suppressor cells; PPARs, peroxisome proliferator-activated receptors; PERK, protein kinase R-like endoplasmic reticulum kinase; EGFR, epidermal growth factor receptor; VEGFR, vascular endothelial growth factor; HER2, human epidermal growth factor receptor 2.

### 5.1 Co-stimulatory molecules

Costimulatory molecules play pivotal roles in regulating T cell proliferation, activation and differentiation. However, inappropriately dampened or enhanced co-stimulatory signals contribute to T cell exhaustion. The activation of T cells relies on both TCR signaling and co-stimulatory signaling from CD28. The effects of CD28 signal are compromised during persistent inflammation, where a significant level of CTLA-4 is expressed. Moreover, the engagement of PD-1 directly inhibits CD28 signaling and prevents T cell activation (88). Critically, CD28 is necessary for successful PD-1 blockade during chronic LCMV infection. Conditional deletion of CD28 has been found to diminish the efficacy of anti-PD-1 therapy in mouse tumor models (89). Continual CD27 signaling has also been reported to result in more severe T cell exhaustion due to excessive T cell stimulation (90). However, another member in the TNF receptor family, 4-1BB, may synergize with anti-PD-L1 to rejuvenate Tex cells (91). The immunoglobulin superfamily member CD226 also plays a role in T cell exhaustion. As a co-stimulatory receptor that mediates T cell adhesion and execution of cytotoxicity (92), CD226 competes with immune checkpoints like TIGIT for the same ligands expressed by cancer cells to execute the anti-tumor effects (93–95). Chronic stimulations on tumor-infiltrating Tex cells induce high expression of Eomes, which downregulates CD226 (67, 96, 97). The Eomes-dependent loss of CD226 limits TCR signaling and anti-tumor effects (98–101). Moreover, the loss of CD226 has been found to impair T cell response to PD-1 blockade (102–104). In a mouse melanoma model, anti-PD-1 therapy was unable to rescue effector functions of Tex cells lacking CD226, implying that the CD226^-^ Tex group displayed a more terminally exhausted profile (105).

### 5.2 Cytokines

Both proinflammatory and immunosuppressive cytokines have regulatory roles in T cell exhaustion. Specifically, high levels of proinflammatory factors trigger the production of unfavorable cytokines which promote T cell exhaustion during long-term infections and malignancies. IL-10 is typically responsive-elevated in inflammatory microenvironments, and blocking IL-10 can prevent and reverse T cell exhaustion (106). The mechanism of reversing exhaustion by blockade of IL-10 may be directly through activating T cells by STAT-3, or indirectly via modulation of suppressive antigen-presenting cells (APCs) (107). In addition, IL-6 and IL-27 are also important cytokines that upregulate co-inhibitory signals on tumor-infiltrating T cells, correlating with T cell exhaustion in both human and mouse tumor models (108–110). The transforming growth factor-β (TGF-β) is another kind of immunosuppressive cytokines that seemingly participated in terminal T cell exhaustion. The expression of TGF-β and the activation of downstream SMAD2 are hallmarks of Tex cells (111, 112). Inhibiting TGF-β1 signaling synergizes with the agonist of bone morphogenetic protein in CD8^+^ T cells *in vitro* enhanced the efficacy of Tex cells (113). However, it was also reported that therapeutic inhibition of TGF-β alone failed to control the persistent LCMV infection and scarcely enhanced the efficacy of antiviral T cells (114, 115).

In contrast, IL-2 responsiveness is crucial for maintaining the Tex cell pool, as IL-2R deficient Tex rapidly disappear during chronic antigen stimulation (116). *In vivo* IL-2 therapy after LCMV chronic infection has been shown to increase the number of antigen-specific Tex and achieve excellent viral control (117). Besides IL-2, recent studies have also indicated that supplying adequate amounts of IL-21 may allow TCF-1^+^ Tpex to escape from exhaustion and differentiate into a CX3CR1^+^ Teff subpopulation that exhibits lower PD-1 and displays improved cytotoxicity compared to Tex (16). What’s more, ambiguous in promoting or preventing T cell exhaustion, type I interferons (IFN-I) play complex roles in controlling T lymphocytes. IFN-I is critical for activating and inducing antiviral functions of CD8^+^ T cells (118), which seems beneficial to avoid T cell exhaustion. However, elevated IFN-I in the tumor microenvironment may also induce high levels of IL-10 and PD-L1 (119). What’s more, it has been reported that IFN-I signaling can promote terminal exhaustion by suppressing Tpex cell sustainment through antagonizing TCF-1 (25). On-going IFN-I exposure has also been linked to the promotion of co-inhibitory receptors (including PD-1, TIM-3, LAG-3, and TIGIT) expression in chronic viral infections (120–124).

### 5.3 Kinases and mTOR signaling pathway

In addition to the co-stimulatory molecules and cytokines that regulate T cell exhaustion, the TCR-linked phosphoinositide 3 kinase (PI3K)-Akt-mTOR pathway also plays an important role in regulating CD8^+^ T cell exhaustion. Upon T cell activation, PI3Kδ inactivates the downstream transcriptional targets FOXO1 and BACH2 to suppress Tcf7 expression (125–127). This results in the repression of TCF-1-mediated central memorization and the up-regulation of effector genes such as *Tbx21* and *Gzmb*, which execute cytotoxicity functions (128–130). Inhibition of PI3Kδ has been reported to promote the expansion of the TCF-1^+^ Tpex subpopulation and improve the response to ICB therapy (131). Tex cells with reduced PI3Kδ activity preserve TCF-1 and promote the generation of a self-renewing memory Tpex population as well (125, 129, 130). Pre-inhibition of Akt or mTOR is also proven to helpfully maintain the stem-like CD8^+^ T cell pool and enhance the antitumor efficacy (132–134). However, it is noticeable that with the exhaustion progressing, mTOR inhibition may no longer limit the chronic viral infection (134), partly because PD-1 signaling weakens the PI3K-Akt-mTOR signaling and causes metabolic reprogramming including impaired glycolysis and mitochondrial activities in late-stage Tex cells (135). So, a more careful modification of this kinase signaling pathway is necessary to balance the stimulation of TCF-1^+^ Tpex cluster properly.

## 6 PD-1 signaling-mediated T cell exhaustion: a fundamental immune adaptation rather than a hindrance to tumor immunotherapy

PD-1 plays an important role in regulating the threshold of antigen response and limiting cytotoxic damage to maintain self-tolerance in T lymphocytes (136). Hence, interrupting the exhaustion program, such as PD-1 blockade, not only can result in enhanced viral control, but also may cause serious immunopathology (137). In fact, immune-mediated pathology is a common side effect of checkpoint blockade in anti-tumor immunity (138, 139). Upon binding with PD-L1/2, PD-1 undergoes a conformational change that brings phosphorylated SHP-2 close to the TCR and CD28 signaling complex (88, 89, 140), resulting in less activation of LCK-mediated ZAP70 and attenuation of downstream RAS-MEK-ERK and PI3K-Akt-mTOR signaling pathways (88). These transduction events ultimately lead to the suppression of T cell proliferation, differentiation, and function execution. This is why higher expression of PD-1 is correlated with more severe T cell exhaustion. Nevertheless, T cell exhaustion should also be considered a physiological adaptation to chronic infections and cancers rather than a deficit in the function of the immune system.

Since chronic infections have existed throughout evolution, T cell exhaustion is a process that avoids immune-mediated pathology while maintaining a degree of functional activity (141). T cells with exhaustion phenotypes can still exert effector functions and mediate the pathogen and tumor control (142–144). CD8^+^ T lymphocytes in established chronic simian immunodeficiency virus (SIV) infections were demonstrated to control viremia efficiently. Virus replication burst rapidly if depleting CD8^+^ T cells in SIV-infected macaques (145, 146). Moreover, a favorable clinical outcome is positively connected with the quantity of tumor-infiltrating CD8^+^ T cells (147). The immune efficacy of CD8^+^ PD-1^hi^ T cells was similar to or even higher than those with less exhausted profiles in secreting cytotoxic molecules (136, 142). Similar phenomena indicate that the effector function of Tex cells is reserved by immune suppressive regulators rather than attenuated intrinsically and irreversibly.

According to this theory, PD-1 stabilizes the TCF-1^+^ Tex precursor cell pool and maintains the survival of this early TCF-1^+^ subset (12). PD-1 activation prevents the loss of TCF-1 expression, and one possible mechanism of PD-1 and TCF-1 interaction is the suppressive effect on TCR and CD28 signaling induced by PD-1 (148). Another theory suggests that PD-1 induces the expression of the transcriptional factor BATF, which positively regulates TCF-1 expression and represses effector-related genes (149). Moreover, BATF has also been predicted as a downstream responder of TCF-1 in the Tpex subpopulation (150). Thus, it is conceivable that a PD-1-BATF-TCF-1 feedback circuit occurs in the Tpex cluster to maintain the early survival of tumor-infiltrating Tex. The Tpex cell cluster is required for an efficient response to ICB therapy because the PD-1^hi^ subpopulation is terminally exhausted and unable to respond to PD-1 pathway inhibition.

PD-1 also functions as an adaptive immune molecule in tumor-specific memory T (Ttsm) cells derived from tumor draining lymph nodes (TdLN). PD-1^+^TCF-1^+^TdLN-residing Ttsm cells do not activate TOX signaling and significantly differ from Tpex and Tex on a transcriptional and epigenetic level (151). Investigations have shown that the TdLN-Ttsm cluster is the primary responder to PD-(L)1 blockade treatment. Blocking PD-L1 effectively amplifies TdLN-Ttsm clusters, resulting in the enlargement of Tpex and Tex in the tumor microenvironment (151). It implies that PD-1 is dispensable for the initiation of T cell exhaustion but it is critical for maintaining the restrained functional state of anti-tumor T cells. To fully understand the impact of anti-PD-1 on various immune cell types, such as B cells, myeloid cells, Treg, and NKs, further investigations are needed. In addition, removing extra immune checkpoints, such as CTLA-4, LAG3, TIM3, and TIGIT) on CD8^+^ T cells might synergistically advance the success of anti-PD-(L)1 therapy (152). However, it is still unclear if there is a mechanism by which inhibitory receptors cooperate, nor is it clear how to optimize interactions between various inhibitory receptors.

## 7 Conclusion

T cell exhaustion is an inevitable outcome of repeated antigen stimulation in chronic infections and tumors. The quantity of Tex cells in the TME is not only a signature for conducting immunotherapy but also a long-lasting “biomarker” for predicting the prognosis. Exploring the heterogeneity of Tex cells reveals advanced therapeutic approaches for reinvigorating T cell exhaustion and improves the efficacy of current ICB therapy. The degree of T cell exhaustion is regulated directly by the intensity and longevity of TCR signaling. Critically, a differentiation hierarchy of T cell exhaustion exists. The progressive transcriptional regulation mechanically orchestrates epigenetic modifications and metabolic reprogrammings to shape distinct Tex clusters. Multiple signal models synergistically contribute to T cell exhaustion, providing many opportunities for combinational treatments to overcome the limitations and achieve success of immunotherapies. Inhibitory receptors play a crucial role in keeping the delicate balance between T cell exhaustion and activation. The best approach to reverse T cell exhaustion still has to be investigated with a considerable understanding of this dysfunctional cell state.

## Author contributions

WT designed the conceptualization. QS, GQ, and XB assisted in the validation. WT and GQ wrote the original draft. QS, WW, and YZ supervised this work. WT performed the visualization. YZ, QS, WC, WW, XB, MJ, YJZ, WL, and HW provided the theoretical support. QS and YZ provided financial support. All authors contributed to the article and approved the submitted version.
